# Costs of hospitalization for stroke from two urban health insurance claims data in Guangzhou City, southern China

**DOI:** 10.1186/s12913-019-4530-2

**Published:** 2019-09-18

**Authors:** Hui Zhang, Yujie Yin, Chao Zhang, Donglan Zhang

**Affiliations:** 1 School of Public Health, Sun Yat-sen University, No. 74, Zhongshan Road 2, Guangzhou, China; 2 Business School, Sun Yat-sen University, No. 135, Xinggang Xi Road, Guangzhou, China; 3 Department of Health Policy and Management, College of Public Health, University of Georgia, 100 Foster Road, Wright Hall 205D, Athens, GA 30602 USA

**Keywords:** Stroke, Cost, Hospitalization, China, Health insurance, Cost of illness

## Abstract

**Background:**

Stroke remains a major global health problem. In China, stroke was the leading cause of death and imposed a large impact on the healthcare system. This study aimed to examine the hospitalization costs by five stroke types and the associated factors for inpatient costs of stroke in Guangzhou City, Southern China.

**Methods:**

This was a prevalence-based, cross-sectional study. Data were obtained from urban health insurance claims database of Guangzhou city. Samples including all the reimbursement claims submitted for inpatient care with the primary diagnosis of stroke from 2006 to 2013 were identified using the International Classification of Diseases codes. Descriptive analysis and multivariate regression analysis based on the Extended Estimating Equations model were performed.

**Results:**

A total of 114,872 hospitalizations for five stroke types were identified. The average age was 71.7 years old, 54.2% were male and 60.1% received medical treatment in the tertiary hospitals, and 92.3% were covered by the urban employee-based medical insurance. The average length of stay was 26.7 days. Among all the hospitalizations (average cost: Chinese Yuan (CNY) 20,203.1 = $3212.1), the average costs of ischaemic stroke (IS), subarachnoid haemorrhage (SAH), intracerebral haemorrhage (ICH), transient ischaemic attack (TIA), and other strokes were CNY 17,730.5, CNY 62,494.2, CNY 38,757.6, CNY 10,365.3 and CNY 18,920.6, respectively. Medication costs accounted for 42.9, 43.0 and 40.4% of the total inpatient costs among patients with IS, ICH and TIA, respectively, whereas for patients with SAH, the biggest proportion of total inpatient costs was from non-medication treatment costs (57.6%). Factors significantly associated with costs were stroke types, insurance types, age, comorbidities, severity of disease, length of stay and hospital levels. SAH was linked with the highest inpatient costs, followed by ICH, IS, other strokes and TIA.

**Conclusions:**

The costs of hospitalization for stroke were high and differed substantially by types of stroke. These findings could provide economic evidence for evaluating the cost-effectiveness of interventions for the treatment of different stroke types as well as useful information for healthcare policy in China.

## Background

As the second most common cause of death worldwide [1], stroke remains a major global health problem [2]. In China, stroke was the leading cause of death in 27 out of 33 provinces by 2013 [3]. Stroke incidence in China is the highest in the world [4]. According to a nationally-representative survey covering 480,687 participants in 2013, the age-standardized prevalence and incidence of stroke reached 1114.8 per 100,000 people and 246.8 per 100,000 person-years, respectively in China [5].

Stroke imposed a huge financial burden on healthcare systems in many countries, consuming about 2–4% of the total health care expenditures across the world [1]. For example, in the United States (US), the country that had the highest medical expenditures in the world, stroke-related annual national spending was estimated to be US$18.8 billion [6]. Comparably, in China, the annual national costs of stroke care was approximately $6 billion [7]. With the population rapidly ageing and the medical costs fast growing, updated information regarding the medical costs associated with stroke is in critical need for health care planning and resource allocation, particularly in middle-and-low-income countries such as China.

Many developed countries have evaluated the direct medical costs associated with stroke, and they used either claims database or hospital discharge data to estimate the medical costs for different types of stroke separately [6, 8–12]. In addition, these studies focused primarily on the inpatient costs, as they remain the largest component of the total direct medical costs associated with stroke [13–15]. For example, in the US, Wang et al. [6] used a national commercial claims database and investigated hospitalization costs by three stroke types (ischaemic stroke (IS), intracerebral haemorrhage (ICH), other strokes). This study found that the average cost of hospitalization (US$20,396) was high in 2008 and varied substantially by these three subtypes of stroke. In Netherland, Buisman et al. [16] used hospital discharge data and conducted a retrospective cost analysis to determine hospital costs of IS and transient ischaemic attack (TIA). They found that inpatient costs were US$6845 for IS patients and US$3173 for TIA patients in 2012. In Singapore, Ng et al. [10] used the National Healthcare Group Chronic Disease Management System database to estimate the direct medical costs associated with stroke and found the types of stroke (subarachnoid haemorrhage (SAH), ICH, IS, TIA) were significantly associated with the costs. Their results showed that the mean direct medical cost was US$10,190 in 2012, while the average cost for patients with SAH was the highest, followed by patients with ICH, IS and TIA. In South Korea, Rha et al. [9] used the reimbursement claims data of the hospital to examine the direct medical costs of stroke by four types of stroke (SAH, ICH, IS, others). They found that the average direct medical cost was US$8732 in 2007, and the cost for ICH was higher than that for IS. In Japan, Yoneda et al. [8] used hospital discharge data and examined the hospital costs of IS and ICH. They reported that inpatient costs for patients with ICH (US$10,260) were higher than those with IS (US$8662) in 2002.

In China, three studies examined the hospitalization costs of stroke [17–19]. Wei et al. [17] used patient interview combined with medical record review to investigate the variations and determinants of hospital costs for acute stroke due to IS or ICH. They found that the overall mean costs of hospitalization were Chinese Yuan (CNY) 11,216 in 2006 and variations in cost were largely attributable to length of hospital stay. Hu et al. [18] used patient interview combined with chart review and estimated the direct medical costs of IS from 300 patients. They showed that the major driver for stroke-related direct medical cost was hospitalization expenses (CNY 18,706.1) in 2010. Jiang et al. [19] used electronic medical system from one hospital and analysed the direct medical costs among 328 hospitalized ICH patients. They reported that the mean direct inpatient cost was CNY 17,028 in 2010. These studies updated the cost estimates associated with stroke in China, however their analyses were limited by the data source and small sample sizes. Furthermore, all three China-based studies were limited to only one or two types of stroke and they did not provide comprehensive information for medical costs categorized by different stroke types. Different types of stroke result in unique disease progression and varied treatment procedures, leading to differences in medical resource use and related costs [20]. As such, hospitalization cost estimation with no consideration of the stroke types may be misleading [6].

Different from the previous literature in China - which either focused on only one or two stroke types or did not use a large-scale insurance claims data, this study aimed to examine hospitalization costs by five stroke types - 1) subarachnoid haemorrhage (SAH), 2) intracerebral haemorrhage (ICH), 3) ischaemic stroke (IS), 4) transient ischaemic attack (TIA) and 5) other strokes using the urban health insurance claims data from the largest city in southern China and investigate the factors that were associated with hospitalization costs.

## Methods

### Data source

Data were obtained from the Urban Employee-based Basic Medical Insurance (UEBMI) and the Urban Resident-based Basic Medical Insurance (URBMI) claims databases of Guangzhou City for the years 2006 through 2013, which contained sociodemographic information, medical conditions and hospitalization costs based on actual payments to providers. The UEBMI (launched in 1998 covering urban employees) and URBMI (launched in 2007 covering the urban unemployed residents) [21] are two social health insurance schemes in China that has been expanded to cover all urban residents. The two insurance schemes have different sources of funding, and the UEBMI scheme offered a more generous benefits with a higher reimbursement rate for many services and more comprehensive service coverage than the URBMI scheme [21]. The most common comorbidities (coronary heart disease, hypertension, diabetes, Alzheimer’s disease, Parkinson’s disease, mental disorder, chronic kidney disease) were identified using personal identifiers from a chronic patient registry under the Outpatient Chronic Disease Program implemented with these two insurance schemes in Guangzhou City. By 2013, 96.6% of the registered residents were enrolled in these two insurance programs in Guangzhou City [22]. This study was approved by the Institutional Review Board of the School of Public Health, Sun Yat-sen University (Approval No. 2017012).

### Study design and patient selection

This was a cross-sectional study conducted in China to estimate the hospitalization costs of stroke. A retrospective, prevalence-based approach was used to determine the medical costs of inpatients with stroke. We obtained all the reimbursement claims submitted for inpatient care with the single primary diagnosis of stroke from 2006 to 2013 using the International Classification of Diseases (ICD) Codes ninth version (ICD-9 used for the years 2006 and 2007) and tenth version (ICD-10 used for the years from 2008 to 2013), which included SAH (ICD-9: 430; ICD-10: I60), ICH (ICD-9: 431; ICD-10: I61), IS (ICD-9: 434; ICD-10: I63, I66), TIA (ICD-9: 435; ICD-10: G45) and other types of stroke (ICD-9: 436–438; ICD-10: I64, I67-I69, 7, 10]. The ICD numbers were coded by professional coders working in the Department of Medical Charts in the hospitals. The last category included poorly defined and sequelae effects of strokes. Patients who were under 18 years old were excluded. In total, 86,126 IS, 1736 SAH, 11,928 ICH, 7298 TIA and 7784 other stroke inpatients were selected. The final sample included 114,872 hospitalizations.

### Cost estimation

The claim databases contained information on the direct medical costs of inpatients with different types of stroke from the healthcare system perspective, including the reimbursement from the health insurance scheme (either UEBMI or URBMI) and out-of-pocket (OOP) payment from the enrolees. The total direct hospitalization costs were separated into laboratory and diagnostic costs, non-medication treatment costs, medication costs, bed fees and the costs of other services, including special caring fees and air-conditioning, based on the classification of costs used in the UEBMI and URBMI schemes. Laboratory and diagnostic costs were the costs of physical examinations and biochemical tests. Medication costs were divided into traditional Chinese medicine and Western medicine. Non-medication treatment costs referred to the costs for any other treatments except for medication, which included blood transfusions, surgery fees, anaesthesia charges, and medical consumables. All costs from 2006 to 2012 in this study were adjusted to 2013 Chinese Yuan (CNY) value using the urban resident’s consumer price index (CPI) of Guangzhou City [22]. The exchange rate between US dollar and CNY was: US $1.00 = CNY 6.2897 in 2013.

### Measures and variables

The outcome variable in the analysis was total hospitalization expenditures per inpatient. The primary independent variable was types of stroke and measured in five categories according to the ICD codes described above: IS, SAH, ICH, TIA, and other strokes. Additional covariates included in the expenditure model were age, gender, insurance type, comorbidities, severity of disease (intensive care unit (ICU) admission, readmission in 15 days and referral from other hospitals), hospital levels (primary, secondary, tertiary), and length of stay (LOS), and years. The information on seven common comorbidities (coronary heart disease, hypertension, diabetes, Alzheimer’s disease, Parkinson’s disease, mental disorder, chronic kidney disease) was matched using personal identifiers with a chronic patient registry under the Outpatient Chronic Disease Program. If not matched, we assumed the patients had no comorbidities. In this study, there were 59,388 patients who had registered to this program.

Those predictors of total hospitalization costs for patients with stroke were chosen in reference to the Andersen’s behavioural model [23] as the conceptual framework. Individual characteristics were identified in terms of: (1) predisposing factors – existing conditions with predispose individuals to use or not use services (e.g. age and gender); (2) enabling factors – conditions that facilitate or impede the use of services (e.g. type of health insurance, hospital levels); and (3) need factors – conditions that healthcare providers recognize as requiring long-term medical treatment (e.g. severity of disease, LOS, comorbidities) [23].

Gender was dichotomized as male vs female, and insurance type was dichotomized as UEBMI vs URBMI. Age was categorized into five groups: 18–44 years old, 45–64, 65–74, 75–79, 80 and older. We used three dummy variables as proxy measures for the severity of disease – whether having an intensive care unit (ICU) admission, having a readmission in 15 days, and having a referral from other hospitals. Hospital level was classified into three levels – primary, secondary and tertiary. The LOS was grouped into three categories: less than 10 days, 10–19 days, 20 days and longer. Comorbidities were measured as binary variables for the following conditions that are available in the registry – whether having a diagnosis of coronary heart disease, hypertension, diabetes, Alzheimer’s disease, Parkinson’s disease, mental disorder, chronic kidney disease. Years were measured as binary variables for controlling the impact of policy changes across the years.

### Statistical analysis

Descriptive statistics (percentage, mean, and standard deviation (SD)) were used for demographic information and costs. Since the value of medical expenditure usually has a skewed distribution, the Kruskal-Wallis test was used to identify the differences in inpatient costs by types of stroke. To identify the predictors of total inpatient costs, the extension of generalized linear model (GLM) - the extended estimating equations (EEE) approach [24] was performed in this study to account for the skewness of cost data. In contrast to the difficulties of selecting the appropriate link function and distribution by the traditional GLM model, the EEE model allows for estimation of flexible link and variance functions using the data at hand, thereby reducing bias and inefficiency in estimation [24]. In order to deal with patients’ rehospitalization, we have corrected the standard errors for clustering at the patient level in the EEE model. The variance inflation factor (VIF) for all predictors used in the model was found to be less than 10 [25], indicating that multicollinearity was not a big problem. In order to address the concern regarding the measurement of comorbidities, we conducted a sensitivity analysis to estimate the predictors of total inpatient costs by limiting the study population only to patients who had comorbidity information available in the registry, and assessed if the assumption of treating the “missing comorbidity” as “no comorbidities” would substantially influence the estimates and the conclusion. All statistical calculations were performed using STATA version 12.0 (Stata Corporation, College Station, TX, USA).

## Results

### Patient characteristics

A total of 114,872 hospitalizations were identified (see Table 1). More than half of the patients were male (54.2%). The average age was 71.7 years old (SD = 11.8). Overall, 40.3% of the patients had hypertension. Most of the patients were under the UEBMI scheme (92.3%) and received medical treatment in tertiary hospitals (60.1%). The mean LOS was 26.7 (SD = 55.3). Only a small proportion underwent hospital referral (1.8%), readmission in 15 days (0.6%) and ICU admission (0.2%).

**Table 1:** Socio-demographic characteristics of inpatients by types of stroke, inpatient data 2006–2013

Characteristics	% or mean ± standard deviation					
	Overall *n* = 114,872	IS *n* = 86,126	SAH *n* = 1736	ICH *n* = 11,928	TIA *n* = 7298	Other strokes *n* = 7784
Gender (%)						
Male	54.2	53.8	44.9	60.8	52.0	52.5
Female	45.8	46.2	55.1	39.2	48.0	47.5
Age (years)	71.7 ± 11.8	72.5 ± 10.9	60.3 ± 17.1	67.1 ± 14.8	71.2 ± 12.1	73.5 ± 11.6
18 ≤ Age < 45	2.4	1.4	16.9	8.0	2.6	1.8
45 ≤ Age < 65	21.8	20.4	38.1	29.4	24.9	18.1
65 ≤ Age < 75	28.2	29.0	21.1	26.0	26.8	25.3
75 ≤ Age < 80	21.4	22.2	12.1	17.0	19.3	23.1
≥ 80	26.3	27.0	11.8	19.7	26.4	31.7
Comorbidities (%)						
None	48.3	46.7	72.3	61.0	45.1	43.8
Coronary heart disease	13.9	14.4	7.0	7.1	19.4	16.3
Hypertension	45.6	46.7	24.9	36.0	47.5	49.9
Diabetes	17.7	18.9	6.6	9.3	17.0	19.9
Alzheimer’s disease	0.7	0.7	0.2	0.5	0.9	0.9
Parkinson’s disease	1.7	1.8	0.4	0.9	1.7	2.1
Mental disorder	0.3	0.3	0.2	0.1	0.3	0.1
Chronic kidney disease	0.4	0.4	0.2	0.4	0.5	0.3
Insurance type (%)						
UEBMI	92.3	92.3	91.7	91.9	93.6	92.4
URBMI	7.7	7.7	8.3	8.1	6.4	7.6
ICU admission (%)	0.2	0.1	2.2	1.0	0.0	0.1
Referral from other hospitals (%)	1.8	1.8	3.5	2.9	0.2	1.3
Readmission in 15 days (%)	0.6	0.6	0.9	1.0	0.3	0.7
Length of stay (days)	26.7 ± 55.3	25.1 ± 52.7	25.2 ± 34.4	41.0 ± 73.2	11.7 ± 11.0	36.9 ± 72.1
< 10	27.0	26.5	29.6	20.0	48.1	22.8
10 ≤ Days< 20	43.1	45.6	29.3	28.9	43.2	41.3
≥ 20	29.9	28.0	41.1	51.1	8.6	36.0
Hospital level (%)						
Primary	9.5	9.5	3.0	7.2	4.3	19.6
Secondary	30.4	29.7	18.7	26.5	33.8	43.3
Tertiary	60.1	60.8	78.3	66.3	61.9	37.1

*IS* Ischaemic stroke, *SAH* Subarachnoid haemorrhage, *ICH* Intracerebral haemorrhage, *TIA* Transient ischaemic attack, *UEBMI* Urban Employee-based Basic Medical Insurance scheme, *URBMI* Urban Resident-based Basic Medical Insurance scheme

Men constituted slightly over half of the patient sample in four stroke subgroups, namely IS (53.8%), ICH (60.8%), TIA (52.0%) and others (52.5%), except for SAH among which 44.9% of the patients were male. SAH is a more severe and life-threating stroke and related with a higher percentage of ICU admission (2.2%) and referral rate (3.5%) than all other types of stroke included. Patients from the SAH subgroup were younger (60.3 ± 17.1) than those from the other four subgroups. On average, patients with ICH tended to have the longest LOS in hospitals (41.0 ± 73.2).

### Direct inpatient costs and cost composition by types of stroke

The mean total direct inpatient cost was CNY 20,203.1 (US $3212.1) for all stroke patients (see Table 2). Among the stroke subtypes, the average costs for inpatients with SAH (CNY 62,494.2) was the highest, followed by ICH (CNY 38,757.6), other strokes (CNY 18,920.6), IS (CNY 17,730.5) and TIA (CNY 10,365.3) (*P* < 0.01). In addition, out-of-pocket (OOP) spending imposes a high burden to stroke patients. The OOP spending accounted for 24.2% of the total hospitalization costs. The OOP expenses among patients with SAH occupied the biggest proportion of total inpatient costs (31.6%) among five types of stroke.

**Table 2:** Direct inpatient costs and cost composition by types of stroke, in Chinese Yuan (CNY)

Composition of total costs	Overall *n* = 114,872	IS *n* = 86,126	SAH *n* = 1736	ICH *n* = 11,928	TIA *n* = 7298	Other strokes *n* = 7784	*P*-Value
Total inpatient costs							
Mean (CNY)	20,203.1	17,730.5	62,494.2	38,757.6	10,365.3	18,920.6	0.000
SD	33,068.4	25,679.9	76,861.2	58,584.3	11,618.4	32,441.7	
Laboratory and diagnostic costs							
Percentage of total inpatient cost (%)	11.0	11.9	6.5	8.9	18.6	7.5	
Mean (CNY)	2215.8	2104.2	4051.2	3453.3	1923.1	1418.4	0.000
SD	2892.0	2480.5	5178.2	4872.2	1604.3	2575.2	
Non-medication treatment costs							
Percentage of total inpatient cost (%)	38.2	36.3	57.6	39.1	34.2	42.6	
Mean (CNY)	7716.2	6441.1	36,001.8	15,136.5	3543.0	8058.6	0.000
SD	16,043.5	12,388.9	50,174.8	24,657.8	7821.1	15,903.3	
Medication costs							
Percentage of total inpatient cost (%)	42.0	42.9	31.4	43.0	40.4	38.5	
Mean (CNY)	8486.3	7600.7	19,615.7	16,672.1	4188.8	7288.0	0.000
SD	14,557.9	11,046.6	28,326.1	28,423.9	4313.5	13,618.8	
Bed fees							
Percentage of total inpatient cost (%)	5.9	6.2	2.4	5.2	5.1	7.8	
Mean (CNY)	1184.3	1093.6	1480.3	2013.6	528.3	1465.7	0.000
SD	2211.2	2037.1	2182.1	3249.6	594.0	2650.1	
Other fees							
Percentage of total inpatient cost (%)	3.0	2.8	2.2	3.8	1.8	3.7	
Mean (CNY)	600.8	490.9	1345.3	1484.6	182.0	689.7	0.000
SD	1798.8	1401.9	2915.7	3555.5	421.9	1717.3	
Out-of-pocket spending							
Percentage of total inpatient cost (%)	24.2	24.1	31.6	23.4	30.5	19.2	
Mean (CNY)	4885.1	4266.7	19,724.5	9059.8	3164.0	3634.2	0.000
SD	8480.3	5969.9	26,910.3	15,317.7	4443.0	6566.6	

*P*-values are based on the Kruskal-Wallis test

*IS* Ischaemic stroke, *SAH* Subarachnoid haemorrhage, *ICH* Intracerebral haemorrhage, *TIA* Transient ischaemic attack

Regarding the cost composition, the biggest contributor of total inpatient costs was medication costs (42.0%). When comparing cost composition across different types of stroke, medication costs accounted for the biggest proportion of hospitalization costs for patients with IS (42.9%), ICH (43.0%), TIA (40.4%). With regards to patients with SAH, the biggest proportion of total inpatient costs was non-medication treatment costs (57.6%).

### Inpatient characteristics associated with inpatient costs by types of stroke

Inpatient costs among different types of stroke significantly differed according to gender, age, comorbidities, insurance type, LOS and hospital level (all *P* < 0.001) (see Table 3). For the entire sample, male patients had higher mean costs of hospitalization than female patients. Younger patients with stroke incurred higher average inpatient costs than older patients. The mean total inpatient costs for UEBMI beneficiaries (CNY 20,369.5) were higher than for URBMI beneficiaries (CNY 18,198.0) on average, and it was true for different types of stroke except for TIA subtype. TIA patients with URBMI had higher inpatient costs than those with UEBMI. When comparing the stroke-related costs among different levels of hospital, the mean inpatient costs in tertiary hospitals was almost twice the amount of those in primary and secondary hospitals.

**Table 3:** Inpatient characteristics associated with inpatient costs by types of stroke

Characteristics	Overall *n* = 114,872		IS *n* = 86,126		SAH *n* = 1736		ICH *n* = 11,928		TIA *n* = 7298		Other strokes *n* = 7784		*P*-Value
	Mean	SD	Mean	SD	Mean	SD	Mean	SD	Mean	SD	Mean	SD	
Gender													0.000
Male	20,379.6	33,581.0	17,708.2	25,907.3	55,231.3	70,584.5	39,796.5	59,798.3	10,588.8	11,559.2	18,639.4	32,766.1	
Female	19,994.2	32,450.2	17,756.6	25,412.6	68,406.2	81,172.7	37,142.9	56,614.4	10,123.5	11,679.0	19,231.5	32,080.8	
Age (years)													0.000
18 ≤ Age < 45	30,830.0	52,130.4	17,372.6	29,601.0	48,820.7	67,059.5	46,814.7	66,701.6	8693.6	9698.5	31,809.3	47,522.5	
45 ≤ Age < 65	21,673.0	38,153.5	16,988.1	26,156.0	71,769.6	78,192.0	42,547.1	64,291.5	10,663.9	12,895.2	18,852.6	34,667.1	
65 ≤ Age < 75	20,149.4	33,619.5	17,577.9	25,935.5	71,247.7	83,822.8	41,099.4	63,406.9	11,621.5	13,461.0	18,804.6	30,168.9	
75 ≤ Age < 80	19,766.1	30,493.6	18,362.4	26,262.8	57,786.0	68,382.5	36,038.6	50,409.9	10,438.1	10,899.3	19,261.2	36,307.7	
≥ 80	18,414.5	26,859.3	17,955.9	24,279.5	41,242.3	74,016.8	29,108.5	42,644.7	8914.9	8394.4	18,063.0	28,460.8	
Comorbidities													0.000
None	21,263.3	36,394.8	17,428.5	26,321.3	60,949.9	77,350.1	40,975.9	61,105.7	10,243.3	12,595.6	20,537.2	36,881.6	
Coronary heart disease	18,815.2	29,022.4	18,425.2	25,276.9	59,525.1	71,116.2	34,598.6	60,429.9	10,240.9	11,614.9	17,755.7	30,522.0	
Hypertension	19,217.4	29,696.6	17,876.4	24,928.1	69,942.3	77,710.7	34,999.0	53,556.6	10,529.0	10,752.4	17,751.1	29,050.9	
Diabetes	19,830.7	28,824.0	19,214.5	26,646.3	57,157.0	62,949.0	37,090.2	51,712.2	11,286.7	11,861.7	18,078.0	27,034.4	
Alzheimer’s disease	17,812.9	23,724.6	17,158.1	19,682.1	25,413.3	20,528.1	31,629.4	50,394.8	10,897.0	9906.6	16,996.4	22,206.7	
Parkinson’s disease	17,332.8	24,084.9	16,733.5	21,101.7	48,605.6	32,399.6	31,354.4	51,948.5	10,834.1	8330.4	17,095.6	24,626.5	
Mental disorder	17,090.6	28,273.8	15,126.3	20,793.6	9951.0	5564.9	80,626.1	117,897.9	12,145.7	5726.6	45,074.8	52,882.0	
Chronic kidney disease	18,372.5	24,761.9	16,817.2	19,381.5	40,562.4	49,982.2	37,708.4	50,887.0	11,104.5	12,977.0	11,435.3	7898.4	
Insurance type													0.000
UEBMI	20,369.5	33,409.0	17,896.0	25,900.0	63,243.1	77,824.9	39,103.3	59,551.1	10,346.8	10,791.7	19,195.3	32,977.4	
URBMI	18,198.0	28,572.3	15,745.0	22,783.0	54,214.3	64,923.4	34,852.4	46,125.8	10,635.0	20,152.0	15,583.1	24,814.0	
Length of stay (days)													0.000
< 10	32,925.6	46,846.2	33,662.4	47,380.6	94,227.6	86,194.9	50,695.3	51,014.9	14,407.9	10,330.2	11,159.9	12,651.0	
10 ≤ Days< 20	12,491.3	10,350.1	17,123.2	31,128.5	46,127.3	37,192.4	37,737.1	85,297.9	5595.6	3774.2	16,015.1	15,019.8	
≥ 20	25,885.1	42,597.4	17,155.1	33,319.7	66,797.6	70,186.4	43,593.0	54,680.7	9483.4	4301.6	30,696.5	39,627.4	
Hospital level													0.000
Primary	14,576.0	24,583.7	13,900.4	23,161.9	25,785.0	61,640.9	24,838.7	36,184.7	4434.5	3891.0	14,156.6	22,423.3	
Secondary	15,078.6	26,023.6	14,073.9	22,389.0	25,835.3	48,718.7	28,850.2	45,977.6	6590.4	6578.0	14,972.1	26,807.3	
Tertiary	23,686.8	36,758.5	20,117.5	27,230.6	72,631.2	79,753.5	44,219.0	64,020.6	12,839.3	13,307.3	26,040.8	40,640.4	

*P*-values are based on the Kruskal-Wallis test

*IS* Ischaemic stroke, *SAH* Subarachnoid haemorrhage, *ICH* Intracerebral haemorrhage, *TIA* Transient ischaemic attack, *UEBMI* Urban Employee-based Basic Medical Insurance scheme, *URBMI* Urban Resident-based Basic Medical Insurance scheme

### Predictors of total inpatient costs

Compared with TIA, the inpatient costs for patients with four other subtypes of stroke were significantly higher, CNY 27,497.1 (SAH), CNY 9655.8 (ICH), CNY 1959.1 (IS) and CNY 848.3 (other strokes), after controlling for other covariates (*P* < 0.01) (Table 4). Compared with the youngest age group (18 ≤ age < 45), the hospitalization costs for older age groups of stroke patients aged over 80 were CNY 817.4 lower after controlling for other factors (*P* < 0.05). The ICU admission, hospital referral and readmission in 15 days were all significantly correlated with higher inpatient costs. Compared with patients with the URBMI scheme, the total inpatient costs of stroke were CNY 910.4 higher than patients with the UEBMI scheme (*P* < 0.01). Patients with a longer LOS and higher hospital levels (e.g., secondary and tertiary level) had significantly higher hospitalization costs (*P* < 0.01). We conducted the sensitivity analysis and found that the subgroup regression results on the cost differences across stroke types from the sensitivity analysis and our total sample regression results reported above were similar, which suggested that the assumption that some comorbidity information might be incomplete was not a concern since it did not significantly affect the cost estimates and did not threaten the robustness of the findings.

**Table 4:** Factors associated with total inpatient costs (EEE model)

	All cases (*n* = 114,872)			*P*-value
	Coef.	Adjusted Std. Err.	Marginal Effect	
Male (Reference: Female)	0.007	[0.004]	131.6	0.050
Age (Reference: 17≤Age < 45)				
45 ≤ Age < 65	−0.012	[0.019]	− 222.3	0.515
65 ≤ Age < 75	−0.019	[0.019]	− 336.8	0.321
75 ≤ Age < 80	−0.028	[0.019]	− 500.7	0.140
≥ 80	−0.046**	[0.019]	−817.4	0.015
Insurance Type (Reference: URBMI)				
UEBMI	0.052***	[0.008]	910.4	0.000
Comorbidities (Reference: None)				
Coronary heart disease	−0.011	[0.005]	−190.4	0.052
Hypertension	−0.049***	[0.004]	− 875.8	0.000
Diabetes	0.035***	[0.005]	638.6	0.000
Alzheimer’s disease	−0.057***	[0.017]	−991.1	0.001
Parkinson’s disease	−0.033***	[0.012]	−588.6	0.005
Mental disorder	−0.039	[0.028]	−687.3	0.166
Chronic kidney disease	−0.005	[0.026]	−93.6	0.843
Stroke Types (Reference: TIA)				
IS	0.111***	[0.007]	1959.1	0.000
SAH	1.024***	[0.037]	27,497.1	0.000
ICH	0.468***	[0.013]	9655.8	0.000
Other strokes	0.047***	[0.010]	848.3	0.000
ICU admission (Reference: None)	1.726***	[0.059]	62,499.3	0.000
Referral from other hospitals (Reference: None)	0.207***	[0.022]	4017.2	0.000
Readmission in 15 days (Reference: None)	0.157***	[0.033]	2989.6	0.000
Length of stay (Reference: < 10)				
10 ≤ Days< 20	0.379***	[0.006]	7244.3	0.000
≥ 20	1.610***	[0.010]	35,417.4	0.000
Hospital level: Primary (Reference)				
Secondary	0.213***	[0.007]	4005.0	0.000
Tertiary	0.700***	[0.010]	11,787.4	0.000
Year: (Reference: Year 2006)				
Year 2007	0.048***	[0.010]	871.6	0.000
Year 2008	0.114***	[0.009]	2110.6	0.000
Year 2009	0.201***	[0.009]	3832.0	0.000
Year 2010	0.265***	[0.009]	5161.1	0.000
Year 2011	0.293***	[0.009]	5681.2	0.000
Year 2012	0.354***	[0.009]	6901.8	0.000
Year 2013	0.369***	[0.011]	7565.7	0.000
λ	0.173***	[0.013]		
θ1	0.752***	[0.013]		
θ2	2.720***	[0.022]		

Standard errors in brackets are adjusted for clustering at the patient level

*IS* Ischaemic stroke, *SAH* Subarachnoid haemorrhage, *ICH* Intracerebral haemorrhage, *TIA* Transient ischaemic attack, *UEBMI* Urban Employee-based Basic Medical Insurance scheme, *URBMI* Urban Resident-based Basic Medical Insurance scheme

*** *p* < 0.01, ** *p* < 0.05. EEE - extended estimating equations model

## Discussion

The present study is an observational study conducted with a large stroke sample in Guangzhou City, the largest and most developed city in southern China. We found that the average cost of hospitalization for stroke patients was CNY 20,203.1 (US $3212.10), while SAH predicted the highest average inpatient costs, followed by ICH, IS, other strokes, and TIA. The types of stroke, insurance types, age, comorbidities, severity of disease, LOS and hospital levels were significantly associated with hospitalization costs of stroke. This is the first study using a large urban health insurance claims database from an entire city to estimate the costs of hospitalization for stroke and compare the inpatient costs among five different stroke subtypes in China.

When comparing our results with findings in other countries, a large variation in costs are observed. The average hospitalization cost (CNY 20,203.1 = US $3212.1) reported in this study was much lower than that in other countries, compared to US$20,396 (2008 price) in the United States [6], €5328 (US$6845, 2012 price) in Netherlands [16], €9004 (US$8104, 2001 price) in Sweden [12], S$12,473.7 (US$10,190, 2012 price) in Singapore [10], US$8732 (2007 price) in South Korea [9] and US$8662 (2002 price) in Japan [8]. Nevertheless, the international comparison of the average healthcare cost for stroke was limited by different estimation approaches (prevalence-based or incidence-based), different types of costs (inpatient or outpatient services), different number of stroke subtypes (one or five subtypes), and different phases of stroke (acute or prevalent stroke, first-ever or recurrent stroke) included in those studies. The variation lies mostly in the different health care systems across countries.

In this study, the mean hospitalization costs of stroke were higher than those reported by three previous studies conducted in China [17–19]. Wei et al. [17] reported that the mean hospitalization cost was CNY 11,216 (2006 price), while CNY 18,706.1 (2010 price) was presented by Hu et al. [18] and CNY 17,028 (2010 price) by Jiang et al. [19]. It is worth noting that none of those studies included all five subtypes of stroke as we did. Wei et al.’s study [17] reported the hospitalization costs of stroke with two subtypes (IS and ICH). Hu et al.’s study [18] analysed the costs only among IS patients, while Jiang et al.’s study [19] only examined ICH patients. Our study included stroke patients with more severe condition (SAH) that may incur higher hospitalization costs, compared to costs reported in previous studies. In addition, Guangzhou City represents a more affluent region of China, and different levels of economic development across geographic regions may explain some of the variations in costs. We used a more recent dataset, and given China’s rapid economic development, it is understandable that our study found higher costs of stroke than previous studies.

Our study is the first study from China that evaluated the hospitalization costs of stroke based on five different subtypes of stroke. Consistent with findings in other countries, our results showed that patients with SAH incurred the highest average inpatient costs (CNY 62,494.2), followed by ICH (CNY 38,757.6), other strokes (CNY 18,920.6), IS (CNY 17,730.5) and TIA (CNY 10,365.3). The regression analysis in this study also suggested that compared with TIA patients, the inpatient costs among patients with SAH, ICH, IS and other strokes were significantly higher. The variation in costs of different subtypes of stroke might be attributable to disease severity and cost composition. Regarding SAH that was reported for the first time in China, the hospitalization cost was the highest, the same as previous studies reported in other countries [9, 10]. In this study, the biggest proportion of total hospitalization costs for SAH patients was spent on non-medication treatment, probably due to a more serious condition such as ruptured intracranial aneurysm, which usually requires neurosurgery and intensive care monitoring [26, 27]. When comparing the costs between ICH and IS, this study found that the inpatient cost for ICH was noticeably higher than the cost for IS, which was in agreement with previous studies in other countries [6, 8, 9]. The higher cost for ICH may be related to the fact that patients in this subgroup are more severe and more often treated by surgery than IS [9]. More severe neurological deficits and more resource utilization may account for longer hospitalization and higher costs in ICH patients [8]. The differences in costs between ICH and IS may be due to the stroke severity, since we found that the ICU admission rate and the mean LOS were higher in patients with ICH than those with IS in this study. With regards to cost composition for these two subtypes, medication costs accounted for the biggest proportion of hospitalization costs for patients with ICH (43.0%) and IS (42.9%). This percentage of medication costs was found to be much higher than patients with IS (4%) in the US [28] and those with IS subtype (12%) in Japan [29], but similar to the findings (41.8% for ICH [19]; 33.6% for IS [18]) in previous China-based studies. The larger proportion of medication costs in China may be due to the higher use of neuroprotectants (e.g., edaravone, ganglioside) and traditional Chinese medicine [17]. In addition, hospital facility costs and provider fees were relatively low in China, also resulting in the relatively higher proportion of medication costs [19]. Among the five stroke subtypes, the hospitalization cost for TIA patients was the lowest. Previous research in other countries [10] also found that patients with TIA had the lowest inpatient costs, probably due to the nondisabling nature of this type of stroke [30]. With minor injuries and better clinical outcomes, TIA patients were more commonly managed in an outpatient setting [30].

Furthermore, the type of health insurance was found to be a significant determinant for the hospitalization costs of stroke in the present study. We found that the UEBMI enrolees with stroke had higher mean costs for hospitalization than the URBMI enrolees for all stroke subtypes except for TIA, mostly because the UEBMI had a higher benefit level for its enrolees [31]. There are two possible explanations for this finding. First, the UEBMI scheme provided more generous benefits with a higher annual reimbursement ceiling, higher reimbursement ratio for many services, and more comprehensive services coverage for its enrollees [21], thereby creating a positive incentive for physicians to provide more comprehensive services and higher quality of care. Second, the URBMI scheme offered neither adequate financial protection nor service coverage for its beneficiaries, thus discourage the URBMI patients to use expensive services [32]. The current health insurance reform to consolidate the different social health insurance schemes will possibly eliminate the barriers that create disparities in benefit designs across health insurance schemes in funding and reimbursement.

Regarding age, younger patients with stroke had higher overall healthcare costs than their older counterparts. Wang et al. [6], together with two related studies [33, 34], also found greater costs for younger adults. As in previous studies [35–38], we found that patients with SAH were much younger than patients with the four other types of stroke including ICH, IS, TIA and others. It serves as an economic rationale that this might be used in the development of prevention programmes. Additionally, the onset of stroke at a young age might incur greater lifetime costs, owing to more medical resource usage, greater recurrence rates [39] and long-term management of diseases [40]. These possibilities could increase the economic burden on the health care system. In addition, males in our study had significantly greater costs than female patients overall, which was the similar result in previous studies [6].

With the help of three proxies (ICU admission, referral from other hospitals, readmission in 15 days) employed in this study to capture the patients’ state of stroke, the severity of stroke was positively related to the inpatient expenditures. The finding was consistent with the previous studies in China and countries outsides of Asia [12, 19]. A history of ICU admission considerably increased the total inpatient costs of stroke, supporting the same conclusion reported in other studies [41]. Comorbidities in stroke patients were also associated with variations in costs. Other countries and China currently face similar stroke risk factors such as hypertension, diabetes, and coronary heart disease. It is worth mentioning that hypertension remains the most important risk factor for all types of strokes with the highest attributable risk at 34.6% [7, 42].

Variations in the costs of different types of stroke tend to be driven by the level of hospitals and LOS, with patients having medical treatment in tertiary hospitals and longer LOS being more likely to incur higher costs of care. Previous studies conducted in China and other countries also found that a longer LOS and hospitalization in tertiary hospitals were positive drivers of inpatient costs for stroke [8, 11, 16, 17]. Tertiary hospitals in China are often better equipped. They provided more precise diagnosis and better medical services but also charged more than secondary hospitals due to higher costs of advanced diagnostic and surgical medical facilities. We identified in this study that the average LOS was 26.7 days, much longer than that reported by academic hospitals in the United States (10.8 days – 19.4 days) [43], and similar to or longer than that in some European countries as well (12 days – 27 days) [44], but shorter than that in Japan (40 days) [8]. The LOS reported in this study was also similar to that in another China-based study (20 days) [17]. The lengthy LOS in China might be due to that most stroke patients tend to stay in hospitals during the post-stroke period. The heavy focus of hospitalization is not cost-effective for the management of chronic diseases such as stroke. Unlike the well-developed stroke systems of care in the US [45], Chinese patients with stroke invested much less in rehabilitation and nursing care (2.3 and 9.8% of the direct cost, respectively) due to a lack of community-based rehabilitation centres or nursing care institutions [18]. Therefore, efforts to establish post-stroke care systems appears to be a cost-saving strategy, which may reduce the burden of health insurance funds and informal caregiving. The findings of this study suggested that strategies to reduce LOS such as building more community-based care facilities along with making changes to the payment models to cover the entire stroke care might be an effective method to contain the costs of stroke.

There were some limitations in this study. First, the study analysed hospitalization costs. The costs of outpatient care, indirect costs due to loss of productivity and family members’ informal care were not examined since our dataset did not include this information. Thus, we likely underestimated the total medical expenditures of stroke in China. Second, clinical severity factors such as neurologic severity, an important predictor of costs, was omitted from the analysis because such data were not available in the claims data. However, we adopted three severity proxies so that stroke severity could be measured. Third, the study population was limited to urban enrolees under two major insurance schemes in one city of China and did not include rural enrolees and people who were not covered in the health insurance system because data were not available, from which the sample cannot represent the whole Chinese population. Fourth, the comorbidity information was linked to another chronic patient registry under the Outpatient Chronic Disease Program, and we had access to the patient registry of seven common chronic diseases (coronary heart disease, hypertension, diabetes, Alzheimer’s disease, Parkinson’s disease, mental disorder, chronic kidney disease). We did not have information on patients of other comorbidities and patients who had these chronic diseases but did not register to this program. Nevertheless, our sensitivity analysis suggested that doing this had no statistically significant impact on our cost estimates, and the missing information due to unmeasured comorbidities would not substantially alter the conclusion of this study. Fifth, the data did not allow us to identify the hospitalizations caused by first-ever or recurrent stroke. Finally, the dataset was a little bit old due to administrative restrictions on data availability, which may not reveal the stroke costs and differences at present. Further studies could consider using a more recent claims dataset, as well as including people covered by all types of insurance, comparing the costs of insured and uninsured patients, and examining the indirect costs for a more comprehensive evaluation of stroke costs.

## Conclusions

The costs of hospitalization for stroke were high and differed substantially by types of stroke. These findings could provide economic evidence for evaluating the cost-effectiveness of interventions for the treatment of different stroke types as well as useful information for healthcare policy in China.

## Data Availability

The datasets used and analyzed during the current study are available from the corresponding author on reasonable request (source: http://www.hrssgz.gov.cn/). Public access to the database is closed.

## Abbreviations

CNY: Chinese Yuan
CPI: Consumer price index
EEE: Extended estimating equations
GLM: Generalized linear model
ICH: Intracerebral haemorrhage
ICU: Intensive care unit
IS: Ischaemic stroke
LOS: Length of stay
OOP: Out-of-pocket
SAH: Subarachnoid haemorrhage
SD: Standard deviation
TIA: Transient ischaemic attack
UEBMI: Urban Employee-based Basic Medical Insurance
URBMI: Urban Resident-based Basic Medical Insurance
US: United States
